# Qualitative Profiling of Venom Toxins in the Venoms of Several *Bothrops* Species Using High-Throughput Venomics and Coagulation Bioassaying

**DOI:** 10.3390/toxins16070300

**Published:** 2024-07-01

**Authors:** Dimoetsha J. C. Weekers, Luis L. Alonso, Anniek X. Verstegen, Julien Slagboom, Jeroen Kool

**Affiliations:** 1 Amsterdam Institute of Molecular and Life Sciences, Division of BioAnalytical Chemistry, Department of Chemistry and Pharmaceutical Sciences, Faculty of Science, Vrije Universiteit Amsterdam, De Boelelaan 1085, 1081 HV Amsterdam, The Netherlands; 2 Centre for Analytical Sciences Amsterdam (CASA), 1012 WX Amsterdam, The Netherlands

**Keywords:** HPLC-MS, high-throughput venomics, *Bothrops* viper venom, coagulation bioassay

## Abstract

Envenoming resulting from snakebites is recognized as a priority neglected tropical disease by The World Health Organization. The *Bothrops* genus, consisting of different pitviper species, is considered the most medically significant taxa in Central and South America. Further research into *Bothrops* venom composition is important to aid in the development of safer and more effective snakebite treatments. In addition, the discovery of *Bothrops* toxins that could potentially be used for medical or diagnostic purposes is of interest to the pharmaceutical industry. This study aimed to employ high-throughput (HT) venomics to qualitatively analyze venom composition while utilizing coagulation bioassays for identifying coagulopathic toxins and characterizing coagulopathic activity in various *Bothrops* venoms. Using the recently demonstrated HT venomics workflow in combination with post-column coagulopathic bioassaying, focus was placed at anticoagulant toxins. Well-known procoagulant toxins were also investigated, taking into account that using the HT venomics workflow, procoagulant toxins are especially prone to denaturation during the reversed-phase chromatographic separations performed in the workflow. The findings revealed that the venoms of *B. atrox* and *B. jararaca* harbored procoagulant toxins, whereas those of *B. alternatus* and *B. neuwiedi* contained both procoagulant and anticoagulant toxins. In general, anticoagulation was associated with phospholipases A_2_s, while procoagulation was associated with snake venom metalloproteinases and snake venom serine proteases. These results showed the identification of coagulopathic venom toxins in the *Bothrops* venoms analyzed using multiple analytical methods that complement each other. Additionally, each venom underwent qualitative characterization of its composition.

## 1. Introduction

Envenoming resulting from snakebites is a public health issue that rural, impoverished areas of tropical countries are mostly affected by and is therefore declared a priority neglected tropical disease by the World Health Organization [1]. The pathological effects resulting from envenomation highly vary, including neuromuscular paralysis (neurotoxicity), coagulopathy (hemotoxicity), and tissue necrosis, as well as swelling and blistering around the bite site (cytotoxicity). The *Bothrops* genus compromises different pitviper species and is considered the most medically significant taxa in Central and South America since this genus accounts for most human envenomings and fatalities compared to any other genus in the region. When it concerns snakebites from a species of the *Bothrops* genus, general symptoms may include thrombosis (blood clotting), hemorrhage (extensive blood loss), and localized necrosis. The variation in pathological effects is a consequence of variation in the toxin composition in snake venom, which occurs in an inter- and intraspecific manner [2]. Snake venoms are complex mixtures containing peptides and proteins known as toxins that snakes use to immobilize and kill prey [3,4]. This wider variety of pharmacological and toxicological effects mostly stems from a diverse range of larger proteins and peptides found in snake venom. Snake venom contains roughly 50–200 components that are distributed in dominant and secondary toxin families and are presented in multiple protein and peptide isoforms. The dominant families include phospholipases A_2_ (PLA_2_s), snake venom metalloproteinases (SVMP), snake venom serine proteases (SVSP), and three-finger toxins (3FTX). The secondary families consist of cysteine-rich secretory proteins, L-amino acid oxidases (LAAO), Kunitz peptides, C-type lectins, disintegrins, and natriuretic peptides. In *Bothrops* venoms, toxins that belong to the dominant families are mostly responsible for their toxicological effects. These toxins mainly come from the PLA_2_, SVMP, and SVSP families of venom toxins [5,6,7]. The only effective treatment that is currently used to counteract envenoming is the intravenous administration of antivenom [1].

Brazilian national laboratories, including the Butantan Institute, Ezequiel Dias Foundation, and Vital Brazil Institute, have successfully developed and mass produced antivenoms using standardized methods in collaboration with the country’s Ministry of Health. These antivenoms were created through horse immunization with pooled venom from one or several medically important venomous snake species living within a certain geographical region for which an antivenom is to be produced, addressing, amongst others, concerns about its effectiveness against venoms from unimmunized *Bothrops* species [8]. The variability in snake venom composition, influenced by factors such as phylogeny, age, sex, geography, and diet, poses challenges to treating snakebite victims [9,10,11]. This variation leads to diverse medical severities, ranging from local tissue damage to life-threatening complications [10]. Additionally, the efficacy of an antivenom may be affected, as antibodies targeting one snake’s venom may be less effective against toxins from other venoms of similar species [2]. Techniques such as proteomics, transcriptomics, and genomics have expanded our understanding of the diverse proteins and peptides in snake venoms. This data allow to identify similarities and differences in venoms from various snakes, considering factors like geography, ontogeny, taxonomy, and sex [5]. Further research on snake species responsible for most human envenomings in the tropical world is crucial to aid in improving current treatments. Also, this knowledge facilitates exploring snake venom toxins for potential medical and diagnostic applications. Next to being the most important venom in terms of pathology in Central and South America, *Bothrops* venoms contain a wealth of different toxins, of which some might be valuable as future drugs or diagnostic tools. The first therapeutic agent developed from a venom in fact was Captopril, derived from the venom of the snake *B. jararaca* [11].

The objective of this study was to utilize HT venomics to comprehensively analyze venom composition in various *Bothrops* species while employing coagulation bioassays to identify coagulopathic toxins and screen their activity. By integrating these methodologies, we aimed to enhance our understanding of *Bothrops* venom complexity from which implications for clinical management and antivenom development could be deduced. Using the recently demonstrated HT venomics workflow [12] in combination with post-column coagulopathic bioassaying as described by Still et al. [13], focus was placed at anticoagulant toxins. Well-known procoagulant toxins were also investigated, taking into account that using the HT venomics workflow, procoagulant toxins are especially prone to denaturation during the reversed phase chromatographic separations performed in the workflow. This study focused on the following species: *B. jararaca*, *B. neuwiedi*, *B. alternatus*, and *B. atrox*. Other recent venomics studies have investigated the venoms of *B. jararaca* [14], *B. leucurus* [15], *B. asper* [12,16,17,18] *B. atrox* [18,19,20,21,22,23,24,25,26], *B. lanceolatus* [21], and *B. brazili* [8,27].

## 2. Experimental

### 2.1. Chemicals and Stock Solutions

The chemicals and solvents used were all analytical grade. Bovine plasma was obtained from Biowest (Amsterdam, The Netherlands). Acetonitrile (ACN; HPLC-R and LC-MS grade) and trifluoroacetic acid (TFA; HPLC grade) were purchased from Biosolve (Valkenswaard, The Netherlands). Water was purified using a Mili-Q plus system from Millipore (Amsterdam, The Netherlands). From Sigma-Aldrich (Zwijndrecht, The Netherlands), pooled snake venom of *B. jararaca*, iodoacetamide, β-mercaptoethanol, ammonium bicarbonate, calcium chloride, and trypsin (proteomics grade, lyophilized powder, recombinant) were sourced. Formic acid (FA) was obtained from VWR (Amsterdam, The Netherlands). Pooled venom of *B. neuwiedi* was sourced from Latoxan (Portes-lés-Valence, France). Pooled venoms from the species *B. jararaca*, *B. alternatus* and *B. atrox* were sourced from the Historic Venom Library at Vrije Universiteit (Amsterdam, The Netherlands). From the freeze-dried venoms stored at –80 °C, 5 mg/mL venom stock solutions were prepared. Aliquots of the reconstituted venom were stored at –80 °C.

### 2.2. High-Performance Liquid Chromatography and Nanofractionation

Optimization of the chromatographic separation for the *Bothrops* venoms under study is given in the Supplementary Materials (SI gradient experiments.docx). The optimal venom concentration for bioassaying was also evaluated. The optimal concentration found was the highest concentration of 5 mg/mL. As the high concentration of venom made its viscosity relatively high, consequently likely lower injection aspiration repeatability during the LC injection step, we continued with 100 µL injection volume at a 1 mg/mL venom concentration. Reversed-phase liquid chromatography (LC) using the optimized LC conditions for HT venomics and for bioassaying was performed on an Agilent LC system (Amstelveen, The Netherlands). Analysis was performed in duplicate for pooled samples of *B. jararaca*, *B. alternatus*, *B. neuwiedi*, and *B. atrox.* Following separation, with a post-column split ratio of 1:9, the larger fraction was directed to a FractioMate FRM100 nanofractionator (VU, Amsterdam, The Netherlands,) under the control of Fractionator software (V1.0, VU, Amsterdam, The Netherlands). The fractions were collected in 384-well plates, with only half of the plates used due to time constraints. To maintain consistency with the Shimadzu HPLC method (50.01 min), the fraction collection time was increased to 12 s per well. The total analysis time on the Agilent LC system was 50.0 min, with a flow rate of 500 µL/min. A total flow rate of 500 µL/min was used. A 4.6 × 100 mm C_18_ column Xbridge Peptide BEH300 (Waters, Etten-Leur, The Netherlands) with a pore size of 300 Å and particle size of 5 µm was used. The temperature of the column oven was set to 40 °C. Mobile phase A comprised 98% MQ, 2% ACN, and 0.1% TFA, while mobile phase B consisted of 98% ACN, 2% MQ, and 0.1% TFA. TFA was selected for its known efficiency in resolving mixtures of peptides and proteins (42). The optimized gradient employed a linear increase of mobile phase B from 1% to 20% over the period between 0.01 and 5 min. At 5 min, there was a gradual increase in % B from 20% to 21.6% over 2 min, followed by an isocratic separation at 21.6% B for 5 min. A sharp increase from 21.6% to 29.6% B occurred at 12 min, taking one minute to complete. Subsequently, at 13 min into the gradient, % B was linearly increased over 17 min to reach 60% B. At 30 min, % B was linearly increased to 90% B over 5 min, followed by an isocratic separation between 35 and 40 min. Within 1 min, % B was decreased back to 1%, and finally, the column was equilibrated for 9 min at 1% B.

The Agilent LC, coupled to an MS (MaXisII qTOF mass spectrometer from Bruker, Bremen, Germany), was used. An electrospray ionization (ESI) source was used with a dry gas flow of 6 L/min, source temperature of 200 °C, and capillary voltage of 3.5 kV. For the control of the instrument and data analysis after a measurement was completed, Bruker Compass software, version 3.0, was used. After fractionation, the plates were evaporated overnight for approximately 16 h using a Christ Rotational Vacuum Concentrator (RVC) (Salm en Kipp, Breukelen, The Netherlands) 2–33 CD plus (Salm en Kipp, Breukelen, The Netherlands). The dried plates were then either stored short-term at −20 °C (further use within a couple days) or stored long-term in a −80 °C freezer. After separation and fractionation, there are two options for further analysis on the dried plates, which are HT venomics and coagulation bioassays. The focus of this study was on HT venomics analysis and coagulation bioassaying of the *Bothrops* venoms included in the study. Therefore, from the MS data measured on the eluted toxins, only the measured Total Ion Currents (TICs) were plotted in the chromatographic Figures in Section 3, for chromatographic comparison with the HT venomics results.

### 2.3. High Throughput Venomics Workflow

**Figure 1:**
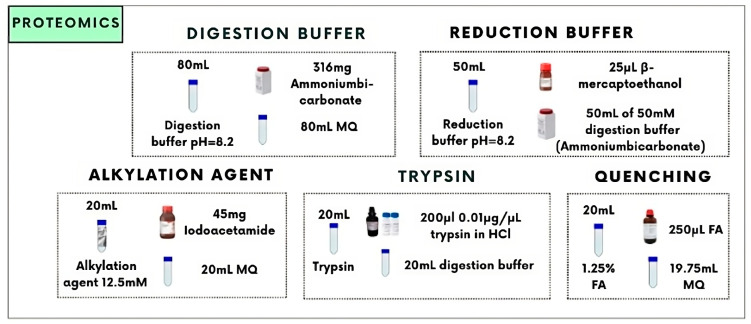
Overview of the protocol for making several solutions for in-solution tryptic digestion used during this research for bottom-up proteomics (i.e., HT venomics). In this figure, the protocol for the preparation of the digestion and reduction buffer, alkylation agent, trypsin, and quenching buffer is given. These solutions are needed for reduction and alkylation using the reduction buffer and alkylation agent, digestion using trypsin, and quenching of the digestion using the 1.25% formic acid (FA) solution.

During this research, the recently published high-throughput (HT) venomics workflow was used. This workflow has first been described by Slagboom et al. [12]. Before HT venomics could be conducted on the dried well plates, tryptic digestion was carried out. The protocol for how the different solutions were made can be found in Figure 1.

**Figure 2:**
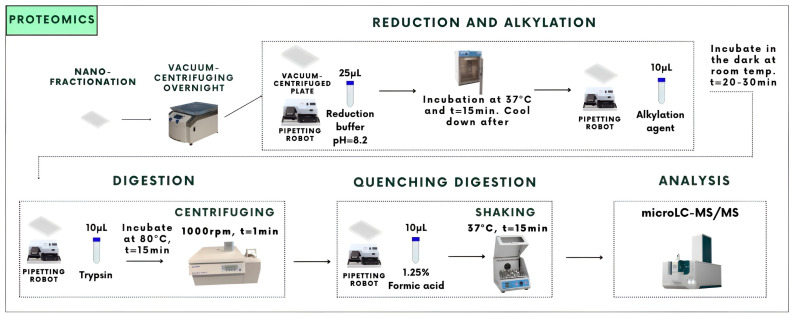
Overview of the protocol for the in-solution tryptic digestion used during this research for bottom-up proteomics (i.e., HT venomics). The digestion was carried out on 384-well plates with fractionated toxins. The fractionation was carried out with the FractioMate FRM100 nanofractionator that was post-column coupled after the chromatographic separation. After the tryptic digestion procedure, the tryptic digests were analyzed by microLC-MS using a ZenoTOF mass spectrometer.

The digestion buffer was made using 316 mg of ammonium carbonate dissolved in 80 mL MQ (pH = 8.2). The reduction buffer contained 50 mL of the digestion buffer and 25 mL β-mercaptoethanol. The alkylation agent was made by dissolving 45 mg of iodoacetamide in 20 mL MQ. In addition, 200 mL of a 0.01 mg/mL solution was added to 20 mL of digestion buffer. Lastly, a quenching buffer was made, which contained 250 mL FA and 19.75 mL MQ. After these solutions were made, the in-solution tryptic digestion was carried out using a pipetting robot and is also visualized in Figure 2.

To each well, 25 mL of the reduction buffer was added. The plates were then incubated at 37 °C for 15 min. After the plates were cooled down, 10 mL of the alkylation agent was added, and the plates were placed in a dark room where they were incubated at room temperature for half an hour. After this step, 10 mL of trypsin digestion solution was added to each well by robotic pipetting using a Thermo Scientific multidrop 384 reagent dispenser, and the well plates were incubated overnight at 80 °C for 15 min. The day after, the plates were centrifuged for 1 min. To quench the digestion, 10 mL of 1.25% formic acid was added to each well using robotic pipetting. After pipetting, the plates were gently shaken for roughly 15 min at 37 °C and were lastly stored at −20 °C until HT venomics measurement.

For the actual HT venomics measurements of the tryptic digests, separation of the tryptic digests took place using an UltiMate 3000 RSLCnano system (Thermo Fisher Scientific, Ermelo, The Netherlands), followed by mass detection on a SCIEX ZenoTOF 7600 system (Nieuwekerk aan den IJssel, The Netherlands). The analysis took 7.2 min for each well. The autosampler was set to an injection volume of 0.2 mL. The gradient used for the separation of the digests was: linear increase from 1% B to 40% at in 4 min followed by a linear increase to 90% in 0.1 min, isocratic separation at 90% B for 1 min, linear decrease to 1% B in 0.2 min, and finally, the column was equilibrated for 2 min at 1% B. The column used for the separation was an analytical capillary C_18_ column Kinetex 2.6 u XB-C18 LC Column (150 mm × 0.3 mm), which was operated at a flow rate of 6 mL/min. The temperature of the column oven was set at 30 °C. After separation, mass detection followed with an ion source operating in positive mode. The method employed a 13 min duration for peptide analysis. Source and gas parameters included curtain gas at 35, CAD gas at 7, ion source gas 1 at 15 psi, and ion source gas 2 at 20 psi, with a temperature set to 150 °C and column temperature to 30 °C. For IDA experiments, a spray voltage of 4.5 kV and a TOF mass range of 400–1500 Da were utilized. Advanced IDA criteria included a mass tolerance of ±50 mDa. TOF MS/MS settings involved fragmentation via CID, with a mass range of 100–2000 Da and a zeno threshold of 100,000 cps. MASCOT was utilized to identify proteins from peptide sequence databases based on the obtained MS and MS/MS data. The MASCOT output MGF files were processed by in-house written scripts as described by Slagboom et al. [12]. This eventually yielded for each venom analyzed an Excel sheet with all processed data that were used for plotting of the Protein Score Chromatograms and which were used to produce the pie charts with qualitative venom compositions presented in this study. These Excel sheets, which contain all processed and raw HT venomics data, can be found in the Supplementary Materials (Processed HT venomics data.xlsx and and zip file Raw HT venomics data).

### 2.4. Post-Column Coagulation Bioassaying in Parallel to the HT Venomics

**Figure 3:**
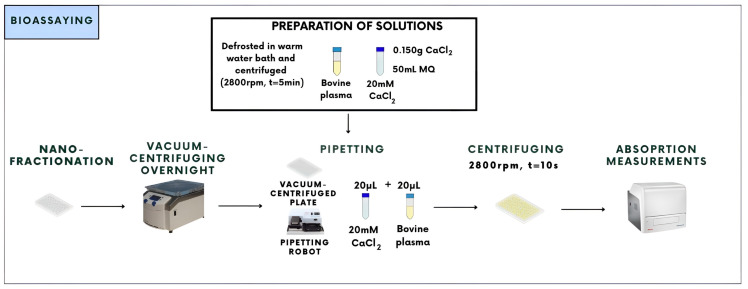
Overview of the coagulation bioassay protocol used during this research, which describes the use of bovine plasma and CaCl_2_ to induce coagulation in 384-well plate format. Well plates contain fractionated and vacuum-centrifuged to dryness venom toxins. After the bioassay solutions are added to the plates, absorption measurements are conducted to assess if there is anti- or procoagulant activity present in wells with fractionated toxins.

Coagulation bioassays on the separated and fractionated venoms were performed according to a bioassay protocol presented in Figure 3.

Before the bioassays could be conducted, bovine plasma was defrosted from either −80 °C or from −20 °C in falcon tubes, using a warm water bath. After defrosting, the plasma tubes were centrifuged to remove any unwanted cellular and other particulate components floating in the plasma. A 20 mM CaCl_2_ solution was made as described Figure 3, for which 0.150 g CaCl_2_ was dissolved in 50 mL MQ. Then, 20 µL of both solutions were transferred into the wells that contain venom fractions. After transferring these solutions into the well plate, the plates were centrifuged for 10 s to remove air bubbles; next, the well plate was quickly inserted into the plate reader (Thermo Fisher Scientific Laboratory Varioskan™ LUX Multimode Microplate Reader using SkanIt 4.1. Measurements) for the absorption measurements. For the absorption measurements, each analysis took around one hour. There was a total of 38 readings that took place at a temperature of 25 °C using a wavelength of 595 nm. This wavelength was chosen because of the yellow color of the bovine plasma. In the context of bioassay data analysis, a distinction was made between fast and slow procoagulation and anticoagulation. If there are bioactive fractions that produce procoagulation, the clotting of the bovine plasma will be faster compared to wells that do not contain fractions with procoagulant bioactivity. Procoagulation results in clotting curves that are faster than normal coagulation, represented by faster increasing absorption in time. In the case of no coagulation at all (i.e., anticoagulation), the baseline of the absorption readout will stay low. Fast procoagulation and slow procoagulation were distinguished by observing clotting early in the measurement (average of the first five readings) or a bit later (average of the first 15 readings), respectively.

In the case of anticoagulation, bioactive fractions will show coagulation that is either reduced or fully inhibited, which, compared to non-bioactive fractions, will show an increased clotting time or no clotting at all. There will be little to no change in absorption in case of full anticoagulation. Anticoagulation was determined as an end point reading, which was reading 38. Procoagulation was plotted as slopes of the coagulation curves. Bioassay chromatograms were finally plotted by plotting on the *x*-axis the retention time of fractionation and on the *y*-axis the bioassay readout, resulting in three bioassay chromatograms for each venom analyzed (i.e., anticoagulation, fast procoagulation, and slow procoagulation).

## 3. Results and Discussion

This research aimed to utilize HT venomics to analyze *Bothrops* venom compositions qualitatively and conduct coagulation bioassays, identifying the toxins in the *Bothrops* venoms analyzed in general and looking into coagulopathic toxins (with a focus on anticoagulation) across the various *Bothrops* species analyzed. Gradient optimization experiments were conducted to ensure effective toxin separation for subsequent UV detection, HT venomics, and coagulation bioassays. Additionally, venom concentration experiments were performed to determine the optimal bioactivity detection concentration, which was found to be an injection volume of 100 µL at a 1 mg/mL venom concentration. Following gradient and venom concentration optimization experiments, four *Bothrops* venoms (*B. alternatus*, *B. atrox*, *B. neuwiedi*, and *B. jararaca*) underwent separation and fractionation for HT venomics and bioassaying. After separation and fractionation, analysis proceeded either with HT venomics for qualitative venom composition determination or through coagulation bioassays to identify procoagulant and anticoagulant toxins. When studying the venom compositions measured by HT venomics, it is important to note that these analyses were qualitative rather than quantitative. HT venomics based on bottom-up proteomics in data-dependent analysis mode returns protein information as sequence coverages and protein scores.

Protein scores were used to obtain indications on relative abundances of toxins in a venom analyzed, but never give real quantitative results as protein scores depend on the peptide scores retrieved of the peptides found back for each venom toxin and these in turn depend on ionization efficiency and fragmentation characteristics. These two parameters are dependent on peptide chemical properties dictated by a peptide’s amino acid sequence and abundance. The key asset of HT venomics is its speed of analysis and straightforward correlation of toxin bioactivities measured using the bioassay with toxin IDs retrieved from HT venomics. While HT venomics thereby provided insights into the general composition of a venom, including the types of toxins present from different toxin families, precise quantification of relative toxin amounts can currently not be achieved by HT venomics. The qualitative compositions of the snake venoms analyzed were further elucidated through pie chart diagrams, visually representing the distribution of toxin families within the venom samples investigated. Bioassays were employed alongside HT venomics to pinpoint coagulopathic toxins present in the *Bothrops* venoms. These bioassay observations were in line with previous research performed on venom from other species [18].

### 3.1. Gradient Optimization Summary

For nanofractionation of the *Bothrops* venoms, an optimized chromatographic gradient was first developed. Detailed information on this optimization process is given in the Supplementary Materials (SI gradient experiments.docx). For the gradient optimization, venoms of *B. jararaca* and *B. neuwiedi* were used. These venoms were primarily selected based on venom stock availability. This study anticipated that the *Bothrops* venoms would predominantly contain metalloproteinases, serine proteases, and PLA_2_s. Initial chromatograms indicated that PLA_2_s eluted between 8 and 10 min, while metalloproteinases and serine proteases eluted between 23 and 35 min. Adjusting the gradient improved resolution, changing elution time profiles for targeted toxins, and notably enhancing separation efficiency of SVMPs and SVSPs by shifting their elution from roughly 23–35 min to 15–32 min. After optimization, the final gradient was also tested on *B. alternatus* and *B. atrox*, from which it was found that toxin separation for these venoms was also good. The final optimized gradient is listed in Section 2. 

### 3.2. Coagulation Bioassays

With the optimized HPLC method, the four venoms under study were separated and fractionated onto 384-well plates for bioassaying. In Table 1, an overview of detected bioactivities is given for each venom, based on the coagulation assay results.

All venoms showed procoagulant activity, while anticoagulant activity was not detected for *B. jararaca* and *B. atrox.* The overall coagulation bioactivity of *B. alternatus* venom was found to be lower compared to the other venoms. The absence of anticoagulant activity for *B. jararaca* and *B. atrox* was unexpected based on the reported presence of PLA_2_s in literature for these venoms [19,20,22,23,24,25,28]. Earlier studies did describe low concentrations of PLA_2_ toxins in *B. atrox* venom, including a study by Sousa et al. [29]. This study gave the determined venom composition of the *B. atrox* investigated and the contribution of PLA_2_s to this *B. atrox venom*. It was shown that the abundance of PLA_2_s was considerably lower (3.2%) than compared to other *Bothrops* venoms like *B. neuwiedi* (8.4%), where anticoagulant activity was indeed detected for. This phenomenon was also observed in this study and will be discussed below.

### 3.3. Integrated HT Venomics, LC-UV and Chromatographic Coagulation Bioassay Results

**Figure 4:**
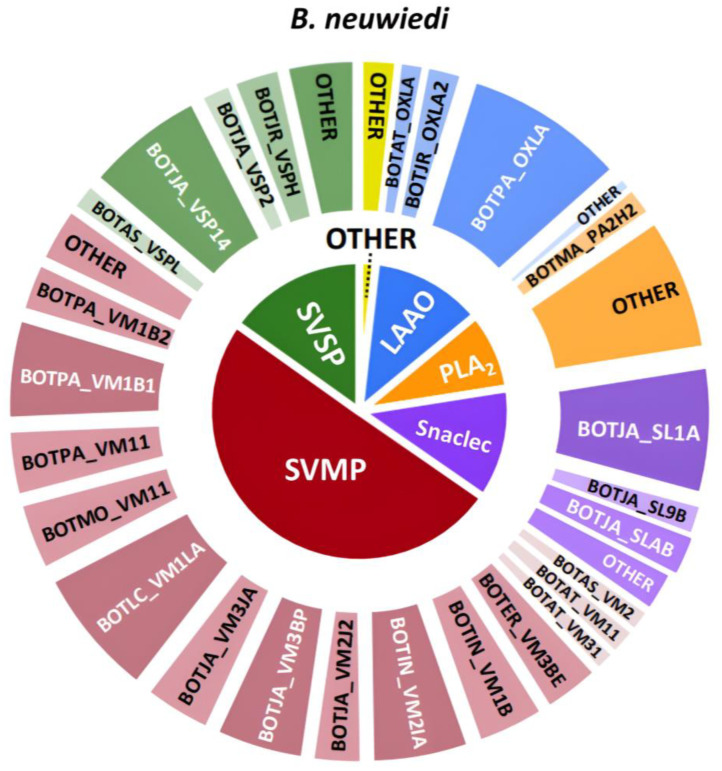
Qualitative contribution of the most prevalent toxin families in the snake venom composition of *B. neuwiedi* is displayed in the inner pie chart. Individual toxins found per toxin family are displayed in the outer pie chart. Qualitative relative abundances are deduced from the qualitative data-dependent analyses performed in the HT venomics approach and do not represent real relative toxin abundances quantitatively. Abbreviations stand for the different toxin families. SVSP: snake venom serine proteinase, LAAO: L-amino acid oxidase, PLA_2_: basic phospholipase A_2_, snaclec: snake venom C-Type lectin, and SVMP: snake venom zinc metalloproteinase. The unique toxins belonging to their respective toxin family shown on the outer pie chart are displayed using their Uniprot protein codes (i.e., resulting from Uniprot database searches by MASCOT). The pie chart was generated using protein scores of toxins with an abundance of >1% in the snake venom and grouping them according to toxin families in Excel and PowerPoint.

In the following section, the venom compositions of all venoms analyzed, including HT venomics, LC-UV, TIC, and chromatographic coagulation bioassay results, are presented in figures in an integrated manner by which the individual chromatographic results are superimposed. In addition, the qualitative venom compositions of *B. neuwiedi*, *B. jararaca*, *B. alternatus*, and *B. atrox* venoms are visualized in separate figures as pie chart diagrams using the HT venomics data. These pie charts were created by assigning total protein scores to each toxin and calculating their contribution to all detected toxins that comprise the overall snake venom composition, deduced from the qualitative data-dependent analyses performed in the HT venomics approach. In practice, the pie charts were produced either manually or using an in-house written script that automatically plots the processed venom toxin data in protein-score based abundances and sorts the toxins found in toxin families. This script, including a manual and example data on producing the pie charts, is given in the Supplementary Materials (Pie chart script.py and PiechartsHTS). Toxins with qualitative abundances higher than 1% were grouped into toxin families, and the total percentages were adjusted to account for the excluded toxins using a calculated factor. This process was performed for all venoms analyzed. Again: It is important to note that these findings are qualitative in nature due to the application of HT venomics. The HT venomics results provide insights into the types of toxins present without precisely quantifying their proportions. In Figure 4, the qualitative venom composition of *B. neuwiedi* can be seen.

**Figure 5:**
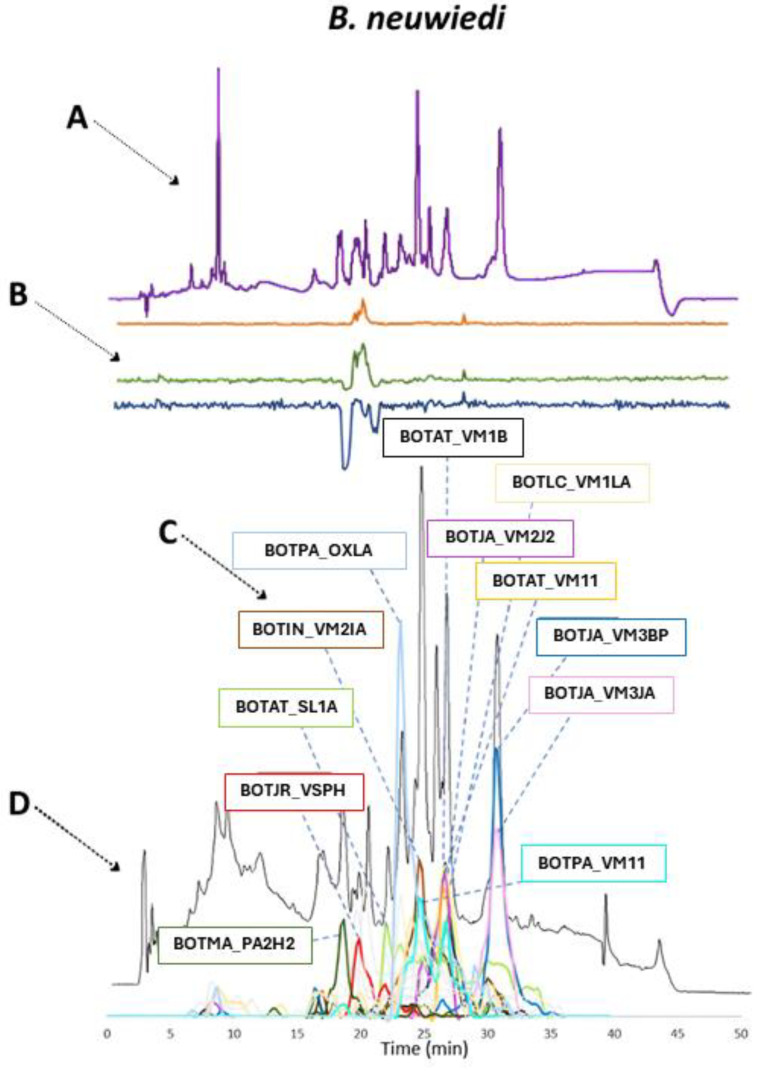
Identification of coagulopathic toxins in the venom of *B. neuwiedi* by correlating several datasets. (**A**) HPLC-UV chromatogram that was measured at 220 nm and is displayed as a purple chromatogram. (**B**) Bioactivity chromatograms that were obtained by conducting coagulation bioassays using bovine plasma and CaCl_2_. The bioassay chromatograms are presented in red, green, and blue. The red and green chromatograms both have positive maxima and represent fast and slow procoagulation, respectively. The blue chromatogram has negative maxima and represents anticoagulation. (**C**) HT venomics data. A selection of identified toxins from the proteomics data are plotted as so-named Protein Score Chromatograms (PSCs) in which, for each identified toxin on the *x*-axis, the retention time of fractionation is plotted, while on the *y*-axis, the protein score of each toxin found in each well is plotted. As all toxins eluted over a series of subsequent wells, these PSCs are the result; (**D**) intact MS data. The black chromatogram displays the total ion current (TIC). The TIC can be used for making peak shape and retention time correlations with the PSCs, the LC-UV, and the chromatographic bioassay data.

Analysis of the venom composition from *B. neuwiedi* suggests a predominant presence of procoagulant metalloproteinases, with their relative abundance constituting roughly 50% of the venom content. Other toxin families, such as serine proteinases, L-amino acid oxidases, PLA_2_s, and snaclecs, appeared to have a similar contribution to the venom composition. Although these findings can only be used qualitatively, it appears that they are in agreement with the earlier discussed bioassay results that describe procoagulation and anticoagulation to be present. How these results compare to the other HT venomics and HPLC-UV data is shown in Figure 5.

**Figure 6:**
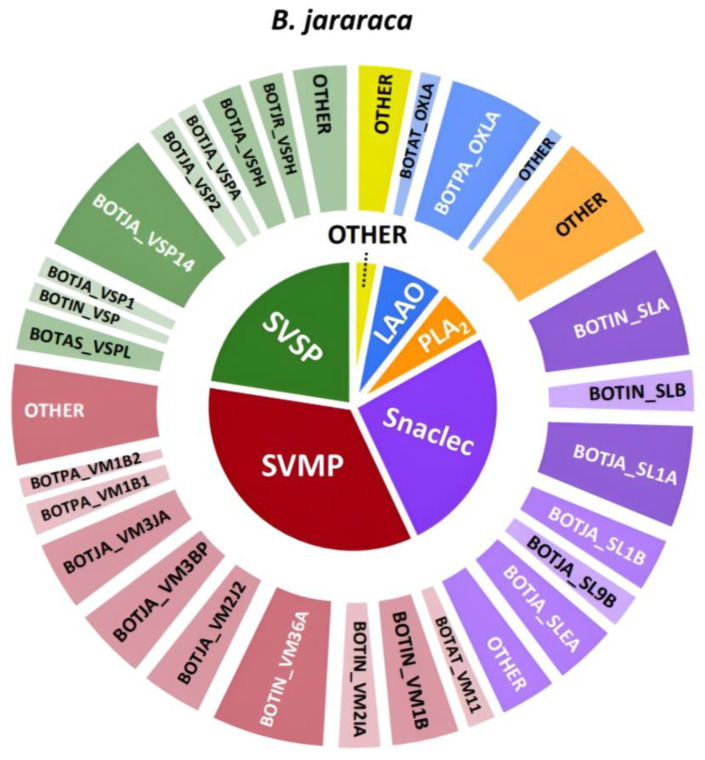
Qualitative contribution of the most prevalent toxin families in the snake venom composition of *B. jararaca* is displayed in the inner pie chart. Individual toxins found per toxin family are displayed in the outer pie chart. Qualitative relative abundances are deduced from the qualitative data-dependent analyses performed in the HT venomics approach and do not represent real relative toxin abundances quantitatively. Abbreviations stand for the different toxin families. SVSP: snake venom serine proteinase, LAAO: L-amino acid oxidase, PLA_2_: basic phospholipase A_2_, snaclec: snake venom C-Type lectin, and SVMP: snake venom zinc metalloproteinase. The unique toxins belonging to their respective toxin family shown on the outer pie chart are displayed using their Uniprot protein codes (i.e., resulting from Uniprot database searches by MASCOT). The pie chart was generated using protein scores of toxins with an abundance of >1% in the snake venom and grouping them according to toxin families in Excel and PowerPoint.

Based on only the overlap in retention times between the HPLC-UV data and the TIC, a total of 12 main toxins could be identified for *B. neuwiedi* from the HT venomics data. Most of these identified main toxins (8/12) belonged to the (possibly procoagulant) SVMPs, which were BOTAT_VM1B, BOTLC_VM1LA, BOTJA_VM2J2, BOTAT_VM11, BOTJA_VM3BP, BOTJA_VM3JA, BOTPA_VM11, and BOTIN_VM2IA. Another possibly procoagulant toxin that could be identified was the SVSP, BOTJR_VSPH. Anticoagulant toxins that were identified were one PLA_2_ (BOTMA_PA2H2) and tentatively one LAAO (BOTPA_OXLA), based on retention time matching of HT venomics with the bioassay data. As explained in the introduction, snaclec toxins can function as either a procoagulant or an anticoagulant [30]. The toxin that was identified, BOTAT_SL1A, can stop von Willebrand factors (vWF) from binding to GPIb. vWF plays an important role in the formation of blood clots and is known to affect blood clotting under high shear stress conditions. When comparing the HPLC-UV data and the TIC with the bioassay chromatograms, it was found that the smaller right peak in the blue anticoagulation chromatogram seemed to correlate with the TIC for BOTAT_SL1A, which implies that this toxin could have anticoagulant activity. For the toxin BOTMA_PA2H2, there was also a correlating anticoagulant peak observed in the bioassay data. For procoagulant activity, fewer toxins could be identified with the use of the bioassay data (BOTJR_VSPH) than expected. Do note that during reversed phase separations of venom toxins, especially the labile proteases, are sensitive to denaturation by the harsh conditions of the eluents (i.e., low pH and high organic solvent concentrations). For procoagulant activity, there was a small window where peaks with positive maxima were identified in. From the HPLC-UV data and the TIC, compatible peaks in terms of retention time were observed, and procoagulant activity was tentatively pinpointed to BOTJA_VM3JA and/or BOTAT_VM1B. The procoagulant toxin that showed the best corresponding results between all datasets was BOTJR_VSPH. The data measured and processed for *B. jararaca* venom is presented next. The qualitative snake venom composition presented as a pie chart is illustrated in Figure 6.

**Figure 7:**
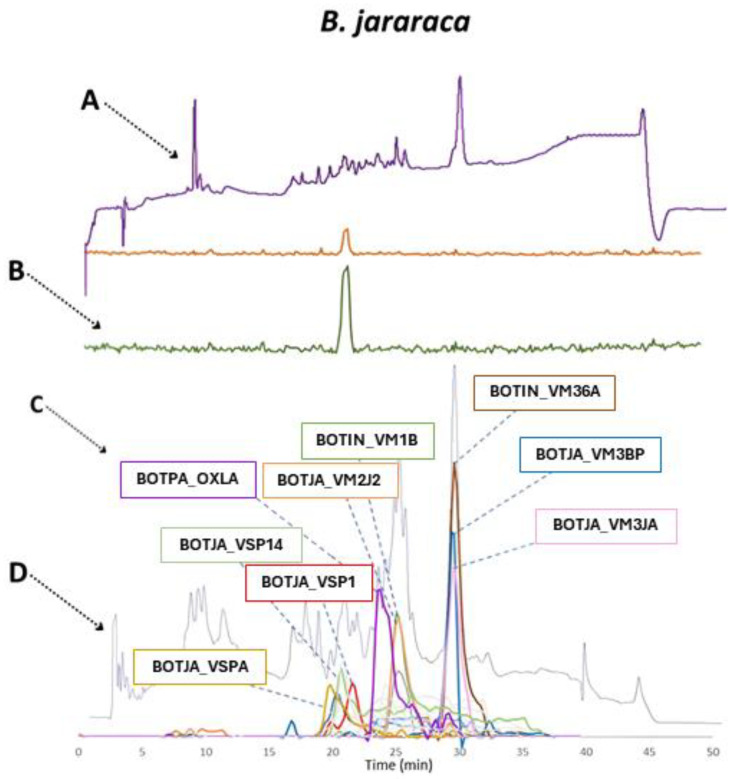
Identification of coagulopathic toxins in the venom of *B. jararaca* by correlating several datasets. (**A**) HPLC-UV chromatogram that was measured at 220 nm and is displayed as a purple chromatogram. (**B**) Bioactivity chromatograms that were obtained by conducting coagulation bioassays using bovine plasma and CaCl_2_. The bioassay chromatograms are presented in red, green, and blue. The red and green chromatograms both have positive maxima and represent fast and slow procoagulation, respectively. The blue chromatogram has negative maxima and represents anticoagulation. (**C**) HT venomics data. A selection of identified toxins from the proteomics data are plotted as so-named Protein Score Chromatograms (PSCs) in which, for each identified toxin on the *x*-axis, the retention time of fractionation is plotted, while on the *y*-axis, the protein score of each toxin found in each well is plotted. As all toxins eluted over a series of subsequent wells, these PSCs are the result; (**D**) intact MS data. The black chromatogram displays the total ion current (TIC). The TIC can be used for making peak shape and retention time correlations with the PSCs, the LC-UV, and the chromatographic bioassay data.

The venom composition of *B. jararaca* mostly resembled that of *B. neuwiedi*, with a notable difference in the higher abundance of snaclecs. Also, metalloproteinases showed a reduced presence as well as PLA_2_s, which are for this venom analyzed by HT venomics grouped under the ‘other’ category due to their insignificant abundance. The very low abundance of PLA_2_s found in the venom analyzed could explain the absence of anticoagulation in the bioassay results. This observation can be seen in the overlaid chromatographic data, which is presented in Figure 7.

**Figure 8:**
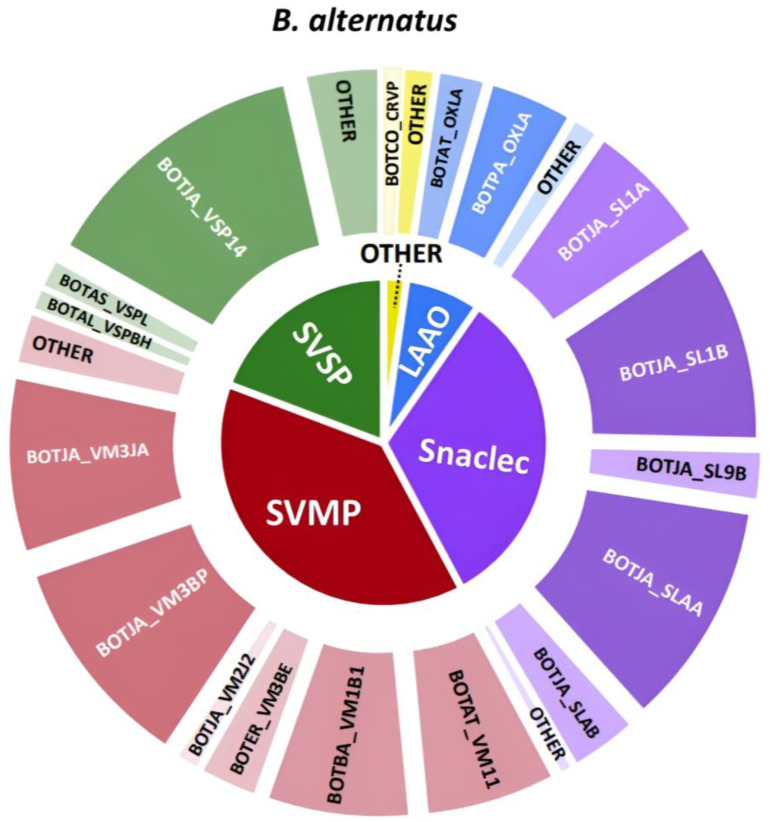
Qualitative contribution of the most prevalent toxin families in the snake venom composition of *B. alternatus* is displayed in the inner pie chart. Individual toxins found per toxin family are displayed in the outer pie chart. Qualitative relative abundances are deduced from the qualitative data-dependent analyses performed in the HT venomics approach and do not represent real relative toxin abundances quantitatively. Abbreviations stand for the different toxin families. SVSP: snake venom serine proteinase, LAAO: L-amino acid oxidase, PLA_2_: basic phospholipase A_2_, snaclec: snake venom C-Type lectin, and SVMP: snake venom zinc metalloproteinase. The unique toxins belonging to their respective toxin family shown on the outer pie chart are displayed using their Uniprot protein codes (i.e., resulting from Uniprot database searches by MASCOT). The pie chart was generated using protein scores of toxins with an abundance of >1% in the snake venom and grouping them according to toxin families in Excel and PowerPoint.

For *B. jararaca*, a total of nine toxins were identified, with eight possibly being procoagulants from the SVMP (BOTIN_VM36A, BOTJA_VM2J2, BOTJA_VM3BP, BOTJA_VM3JA, and BOTIN_VM1B) and SVSP family (BOTJA_VSP14, BOTJA_VSP1, and BOTJA_VSPA). From the bioassay data, no anticoagulation peaks were observed (and hence the anticoagulation chromatogram was not plotted in the figure). As was mentioned before in the discussion of the bioassay results, there was no anticoagulant activity detected for *B. jararaca*, which was unexpected. The HT venomics data also were unable to identify any likely potent toxin with anticoagulant activity, which supports the hypothesis that potent anticoagulants are not or only present in *B. jararaca* venom in a low concentration. BOTPA_OXLA is a highly abundant (but having a low anticoagulation potency) LAAO, with known anticoagulant activity [31,32,33]. For procoagulant activity peak observed in the bioassay chromatogram, the TIC and UV data were compared. It was concluded that BOTJA_VSP1 could be responsible for this bioactivity. In the *B. jararaca* dataset, it was also evident that bioactivity peaks were only visible in a narrow time window in the bioassay chromatograms, while additional possibly coagulation modulating toxins were detected outside of this window in the HT venomics dataset. When comparing the different datasets, it was concluded that there was a main agreement between the UV and bioassay data, the TIC data, and the HT venomics data for the procoagulant activity of toxin BOTJA_VSP1. The two last venoms analyzed that will be discussed are that of *B. alternatus* and *B. atrox*. The qualitative venom composition findings presented as a pie chart for *B. alternatus* are displayed in Figure 8.

**Figure 9:**
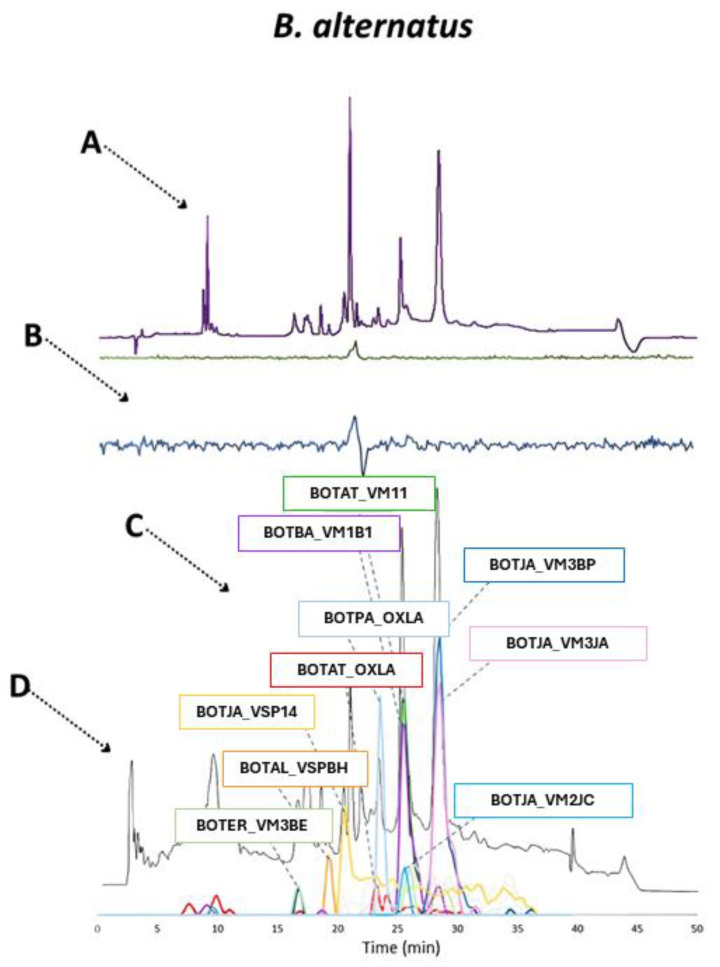
Identification of coagulopathic toxins in the venom of *B. alternatus* by correlating several datasets. (**A**) HPLC-UV chromatogram that was measured at 220 nm and is displayed as a purple chromatogram. (**B**) Bioactivity chromatograms that were obtained by conducting coagulation bioassays using bovine plasma and CaCl_2_. The bioassay chromatograms are presented in red, green, and blue. The red and green chromatograms both have positive maxima and represent fast and slow procoagulation, respectively. The blue chromatogram has negative maxima and represents anticoagulation. (**C**) HT venomics data. A selection of identified toxins from the proteomics data are plotted as so-named Protein Score Chromatograms (PSCs) in which, for each identified toxin on the *x*-axis, the retention time of fractionation is plotted, while on the *y*-axis, the protein score of each toxin found in each well is plotted. As all toxins eluted over a series of subsequent wells, these PSCs are the result; (**D**) intact MS data. The black chromatogram displays the total ion current (TIC). The TIC can be used for making peak shape and retention time correlations with the PSCs, the LC-UV, and the chromatographic bioassay data.

*B. alternatus* does not seem to have a significant amount of PLA_2_ and was mostly dominated by metalloproteinases and snaclecs based on the qualitative HT venomics results, and it had little anticoagulant activity as seen in the bioassay results (Figure 9). What is interesting is that there is clearly a small difference in terms of the number of significant toxins found for each toxin family compared to the other two venoms analyzed and discussed above. These results seem to make this venom appear less complex. Although there were fewer individual toxins found, the contribution of each toxin family to the venom was still similar as that of the *B. jararaca* and *B. neuwiedi* venom. The overlaid chromatographic datasets are displayed in Figure 9.

**Figure 10:**
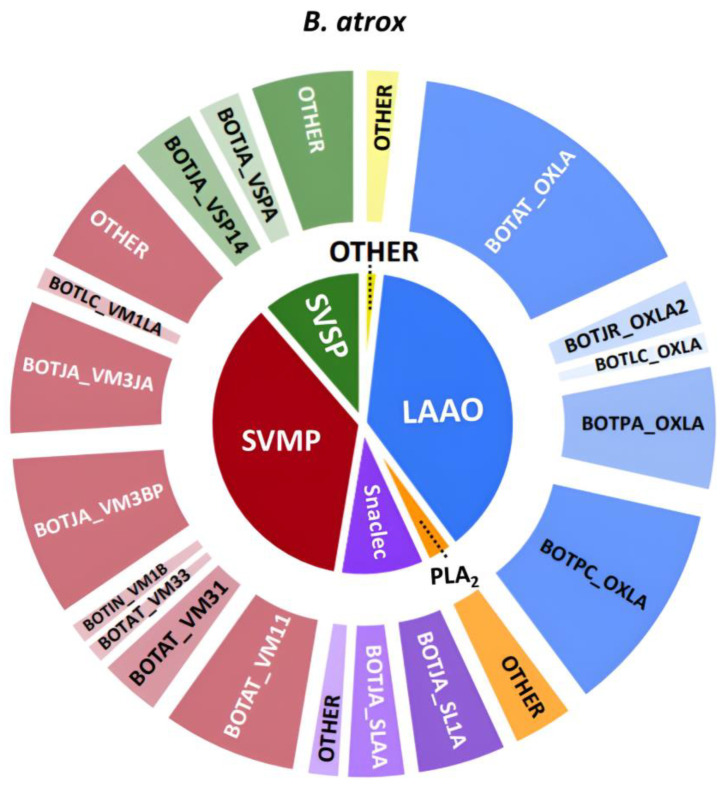
Qualitative contribution of the most prevalent toxin families in the snake venom composition of *B. atrox* is displayed in the inner pie chart. Individual toxins found per toxin family are displayed in the outer pie chart. Qualitative relative abundances are deduced from the qualitative data-dependent analyses performed in the HT venomics approach and do not represent real relative toxin abundances quantitatively. Abbreviations stand for the different toxin families. SVSP: snake venom serine proteinase, LAAO: L-amino acid oxidase, PLA_2_: basic phospholipase A_2_, snaclec: snake venom C-Type lectin, and SVMP: snake venom zinc metalloproteinase. The unique toxins belonging to their respective toxin family shown on the outer pie chart are displayed using their Uniprot protein codes (i.e., resulting from Uniprot database searches by MASCOT). The pie chart was generated using protein scores of toxins with an abundance of >1% in the snake venom and grouping them according to toxin families in Excel and PowerPoint.

As was mentioned earlier in the bioassay’s results section, it was assumed that the venom of *B. alternatus* has lower coagulation modulating activity compared to the other *Bothrops* venoms analyzed, based on the analytical approach used in this study. The main toxins found in the HPLC-UV and TIC data corresponded to the SVMPs BOTJA_VM3JA, BOTJA_VM3BP, BOTJA_VM2J2, BOTAT_VM11, BOTAT_VM1B1, and BOTER_VM3BE. There were also SVSPs identified, such as BOTAL_VSPBH and BOTJA_VSP14. There were a limited number of small peaks detected in the bioassay data compared to the other two datasets discussed above. Although there was no real matching procoagulant toxin pinpointed that matched across all datasets, the toxin BOTJA_VSP14 was identified as possible matching procoagulant toxin based on the UV, HT venomics and TIC plots. This peak could, however, not be identified in the bioassay data, which is probably due to its likely relatively low abundance in the venom analyzed. The last dataset that will be discussed is that of *B. atrox*, for which the qualitative HT venomics results represent as a pie chart is given in Figure 10.

In the case of *B. atrox*, the most prevalent toxin families found in the venom were metalloproteinases and L-amino acid oxidases, which differ from the anticipated venom composition. Likely, the LAAO contribution in the venom composition measured by HT venomics was strongly overestimated in this analysis. From experience using HT venomics, LAAOs are, in many cases, overrepresented in terms of qualitative abundances found. This is likely due to the good sensitivity of proteomics analysis of many of the tryptic peptides of LAAOs, thereby yielding high protein scores, even if LAAOs are present in a relatively low concentration compared to other toxin classes such as SVMPs and SVSPs. Previous studies evaluating the venom composition of *B. atrox* in the Brazilian Amazon region reported a predominance of metalloproteinases, accounting for approximately 50% of the venom, followed by snaclecs, serine proteases, and a smaller percentage of PLA_2_, cysteine-rich secretory proteins, and L-amino acid oxidases [22]. Another study examining *B. atrox* venom from specimens in the Peruvian Amazon indicated that the L-amino acid oxidase content constituted 10.5% of the total protein content [23].

**Figure 11:**
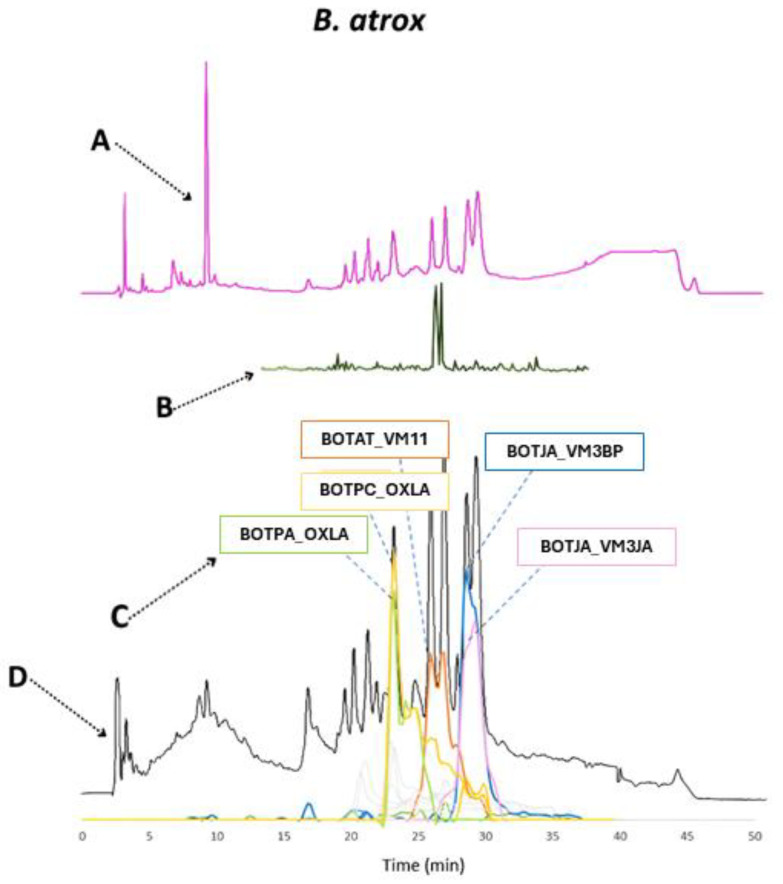
Identification of coagulopathic toxins in the venom of *B. atrox* by correlating several datasets. (**A**) HPLC-UV chromatogram that was measured at 220 nm and is displayed as a purple chromatogram. (**B**) Bioactivity chromatograms that were obtained by conducting coagulation bioassays using bovine plasma and CaCl_2_. The bioassay chromatograms are presented in red, green, and blue. The red and green chromatograms both have positive maxima and represent fast and slow procoagulation, respectively. The blue chromatogram has negative maxima and represents anticoagulation. (**C**) HT venomics data. A selection of identified toxins from the proteomics data are plotted as so-called Protein Score Chromatograms (PSCs) in which, for each identified toxin on the *x*-axis, the retention time of fractionation is plotted, while on the *y*-axis, the protein score of each toxin found in each well is plotted. As all toxins elute over a series of subsequent wells, these PSCs are the result; (**D**) intact MS data. The black chromatogram displays the total ion current (TIC). The TIC can be used for making peak shape and retention time correlations with the PSCs, the LC-UV, and the chromatographic bioassay data.

There were mainly five toxins identified in *B. atrox* (see Figure 11), namely the (likely procoagulant) SVMPs BOTAT_VM11, BOTJA_VM3BP and BOTJA_VM3JA, and the (likely anticoagulant) BOTPC_OXLA and BOTPA_OXLA. For *B. atrox*, a clear correlation across the different datasets can be seen for procoagulant toxin BOTAT_VM11.

As snake venom plays a crucial role in the ecological functioning of venomous snakes, its composition changes due to the evolutionary history of a species and how they adapt to their environment using specific toxins [34]. Viperid venoms are extremely complex and varied but are composed of related toxin isoforms that only present a handful of protein families. Andrade-Silva et al., for example, found that post-translational glycosylation was largely responsible for intraspecific venom variation within the *Bothrops* genus based on qualitative and quantitative analysis of venom [35]. What was seen in this study for all *Bothrops* venoms and has been highlighted several times in this study is that there only seems to be bioactivity within a narrow time frame (from 15 to 20 min) in the bioassay chromatograms, while toxins seem to be identified across a broader time frame range when inspecting the UV, TIC, and HT venomics PSC data. Between 15 and 32 min, there seemed to be multiple peaks overlapping across the different datasets, which makes it highly unlikely that there were no other toxins with procoagulant activity as SVMPs and SVSPs were eluting within this time window. Why coagulant activity could only be identified within a narrow time frame for all bioassays was most likely due to toxin denaturation during the chromatographic separations. To overcome this issue, currently, we are implementing analytical non-denaturing ion exchange (IEX) and hydrophobic interaction (HIC) chromatographic separations prior to HT venomics and bioassaying. The main bottleneck to overcome in employing this way of analysis is the incompatibility of high concentrations of nonvolatile salts used for IEX and HIC, especially with mass spectrometry, but also with post-column bioassaying. This work, however, is out of scope for the current study and will be presented in future research from our group. The main achievement in this study is successfully presenting the qualitative venom composition of the *Bothrops* venoms analyzed next to correlating toxin IDs with bioassay, LC-UV, and TIC data.

The HT venomics data indicate a significant similarity in the toxin families present in the venoms of *B. jararaca*, *B. neuwiedi*, *B. alternatus*, and *B. atrox*. These venoms primarily consisted of SVMPs, SVSPs, LAAOs, snaclecs, and PLA_2_s. The qualitative distribution of these toxins was generally similar among these species, with the exception of *B. atrox*, which showed a notably higher abundance of LAAOs and SVMPs. In contrast, the other species exhibited a higher abundance of SVMPs, SVSPs, and snaclecs. In our study, venomics and coagulation bioassay data suggested that the observed procoagulant activity was largely due to the abundant presence of SVSPs and SVMPs. Conversely, the anticoagulant activity was likely caused by the presence of PLA_2_s. These data provide insights into the bioactivity of the identified toxins. The identified abundant toxin families and distribution of venom components observed in our study aligns with findings from other venomics research [8,12,14,15,16,17,18,19,20,21,22,23,24,25,26,27,29]. One such study analyzed *Bothrops* venoms using shotgun nanoESI-LTQ/Orbitrap analyses and identified 15 different protein groups in various proportions. The analyses revealed that SVMPs were the most abundant toxins in all venoms, especially in *B. atrox*, *B. alternatus*, and *B. jararaca*. Significant amounts of PLA_2_s were found in *B. neuwiedi* venom. Additionally, a notable presence of snaclecs was detected in the venoms of *B. jararaca*, *B. alternatus*, and *B. atrox*. SVSPs and LAAOs were almost equally distributed among all the venoms analyzed [16]. The biggest difference compared to other studies is that our approach enables the rapid identification and pinpointing of procoagulant and anticoagulant toxins in venoms using HT venomics in combination with integrated bioassays. These toxins can be examined further in greater detail using conventional low-throughput bioassay technologies. Instead of using literature to establish the possible coagulant activity of identified toxins, the here-presented method makes it possible to directly correlate coagulopathic toxin peaks with toxin IDs, since our approach to venomics allows for the plotting of PSCs, and correlate them with coagulopathic bioassay peaks from the bioassay chromatograms.

## 4. Conclusions

The objective of this study was to utilize HT venomics to qualitatively analyze venom composition of various *Bothrops* species. In addition, employing coagulation bioassays to identify coagulopathic toxins was demonstrated. By integrating these methodologies, we aimed to enhance our understanding of *Bothrops* venom complexity. These results might aid in the clinical snakebite management and antivenom development. HT venomics data were obtained from four pooled venoms, from *B. atrox*, *B. alternatus*, *B. jararaca*, and *B. neuwiedi* snakes. Matching HT venomics data, bioassay data, and UV data revealed agreements between some peaks of the abundant toxins and bioassay peaks. This approach of peak shape and retention time matching of datasets increases the confidence of (bioactive) toxin identification. It became clear that the absence of anticoagulant peaks in the bioassays of *B. jararaca* and *B. atrox* was also evident from the HT venomics results. In these venoms, anticoagulant toxins likely seemed to be less prevalent compared to the other venoms analyzed. Overall, all venoms analyzed had the same composition in terms of toxin families found, but with varying abundances. A majority of the identified proteins for all venoms came from similar species as those actually analyzed. This discrepancy can be attributed to the limited amount of data available for protein identification for the species that were actually analyzed. Procoagulant activity in the venom of *B. neuwiedi* was most likely caused by SVSPs and anticoagulant activity by PLA_2_s and possibly snaclecs. For *B. jararaca*, procoagulant activity could be assigned to the presence of SVSPs such as BOTJA_VSP1. A possible toxin that could have caused the anticoagulant activity observed in the bioassays could not be identified. The venom of *B. alternatus* showed low coagulation modulating activity compared to the other *Bothrops* venoms. For *B. atrox*, toxin BOTAT_VM11, which is an SVMP, was identified as procoagulant toxin. What is interesting is that there were no abundant SVSPs identified. This venom also comprised a high abundance of LAAOs, likely overrepresented in the venom analyzed due to the probable average good sensitivity of analysis of the tryptic peptides coming from LAAOs after the tryptic digestion step in the bottom-up proteomics-based HT venomics approach. Based on these results, it can be concluded that the general snake venom compositions of several *Bothrops* venoms were successfully characterized qualitatively using parallel integrated analytical methods.

## Figures and Tables

**Table 1:** Overview of the biological activities (i.e., procoagulation and/or anticoagulation) found for all *Bothrops* snake venoms investigated.

Species	Fast Procoagulation	Slow Procoagulation	Anticoagulation
*B. neuwiedi*	Yes	Yes	Yes
*B. jararaca*	Yes	Yes	No
*B. alternatus*	No	Yes	Yes
*B. atrox*	No	Yes	No

## Data Availability

The high-throughput venomics data used for plotting the PSCs is provided in the Supporting Information.

## Supplementary Materials

The following supporting information can be downloaded at: https://www.mdpi.com/article/10.3390/toxins16070300/s1. Word document S1: Gradient experiments; Zip file S1: Raw HT venomics data; Excel document S2: Processed HT venomics data; Script S1: Pie chart script.py and Script S2: PiechartsHTS. References [3,12,20,25,26,28,29,30] are cited in the Supplementary Materials.
