# Supplementary material for: Qualitative Profiling of Venom Toxins in the Venoms of Several Bothrops Species Using High-Throughput Venomics and Coagulation Bioassaying
Source: Toxins (Basel). 2024 Jul 1;16(7):300. doi: 10.3390/toxins16070300 (PMC11280908; doi:10.3390/toxins16070300)
Supplement: Supplementary file 1 [file toxins-16-00300-s001.zip › Supporting information gradient experiments.pdf]

Supporting Information: Qualitative profiling of venom toxins in the venoms of several *Bothrops* species using high throughput venomics and coagulation bioassaying

High performance liquid chromatography for chromatographic optimization

Before running the bioassays and HT venomics under optimal conditions, the separation of Bothrops venoms was optimized. This was done using a Shimadzu HPLC system ('s Hertogenbosch, The Netherlands). For the separation, a gradient that was previously used for the separation of viper venoms by our group was optimized for the Bothrops genus (30). The optimization of the gradient was performed using a trial-and-error approach. Based on prior research, it was already known which toxin families would likely dominate the Bothrops venoms and in which order they were expected to elute [29,36–40]. To enhance the resolution of peaks in the time frame where specific toxins were expected to emerge, adjustments were made to the organic modifier percentage (%B). This allowed the fine-tuning of the retention of analytes on the stationary phase, thereby improving separation. When analytes were insufficiently separated from one another, we determined the %B value at which they would elute from the stationary phase and then slightly extended the %B at that time to slightly extend elution time of these toxins. To maintain the same overall analysis time, the %B values at which no relevant analytes appeared to elute were pinpointed and the %B percentages at these time frames were linearly increased swiftly, thereby reducing analysis time at these time frames. Various modifications of this nature were implemented in the experimental gradients, and we compared the resulting chromatograms to evaluate the impact of these subtle alterations on peak resolution. Prior to entering the chromatographic column, the venom samples underwent injection into the system. After separation following a post-column split ratio of 1:9, the larger aliquot was directed to the nanofractionator. Meanwhile, the smaller aliquot was routed for UV detection utilizing a Shimadzu SPD-M30A photodiode array, with detection set at 220 and 254nm. Chromatographic settings were controlled using Shimadzu LabSolutions software for the HPLC. The two Shimadzu pumps were set to a total flow rate of 500 µl/min. A 4.6x100mm C_{18} column (Xbridge Peptide BEH300) with a pore size of 300Å and particle size of 5µm was used. The temperature of the column oven was set to 40°C. Mobile phase A was comprised of 98% MQ, 2% ACN and 0.1% TFA. Mobile phase B was comprised of 98% ACN, 2% MQ and 0.1% TFA. Venoms at different concentrations were prepared for the initial screening using this setup to find the concentration where bioactivity could be optimally detected. For pooled venoms of *B. Jararaca*, *B. Alternatus* and *B. Neuwiedi* samples, concentrations of 1, 2.5 and 5 mg/mL were prepared. Additionally, venom concentration experiments were performed on the Shimadzu HPLC-UV for several venoms (*B. Alternatus*, *B. Neuwiedi*, and *B. Jararaca*) at 1, 2.5, and 5 mg/mL with an injection volume of 20µL to determine the optimal bioactivity detection concentration. Due to limited resources, only samples of 1 mg/mL were prepared for *B. Atrox*. Measurements were done in duplicate. 20uL venom of each concentration was analyzed. Only the optimal venom concentrations analyzed are shown in this study. The optimal concentration found was the highest concentration of 5 mg/ml. As the high concentration of venom made its viscosity relatively high with as consequence likely lower injection aspiration repeatability during the LC injection step, we continued with 100µl injection volume at a 1 mg/ml venom concentration.

Supporting Information: Qualitative profiling of venom toxins in the venoms of several *Bothrops* species using high throughput venomics and coagulation bioassaying

Gradient optimization

A gradient that was previously used for the nanofractionation of viper venoms was optimized for the nanofractionation of the *Bothrops spp.* venoms included in this study to increase separation efficiency of the toxins in the Bothrops venoms to be analyzed. The final optimized gradient, an alternative gradient to the optimized gradient, and the original viper gradient [17] will be compared to demonstrate the effect of increasing the percentage of organic modifier (i.e., eluent B) at a slower or faster pace on the resolution and separation efficiency of the separated peaks for the different venoms analyzed. For the gradient optimization, venoms of *B. Jararaca* and *B. Neuwiedi* were used. These venoms were primarily selected based on stock availability. After optimization, the final gradient was tested on the remaining venoms from the *Bothrops spp.* genus included in this study (from which it will be shown that separation was proven to be good). In Figure 1 the eluent B percentage plots of the three above mentioned gradients can be seen.

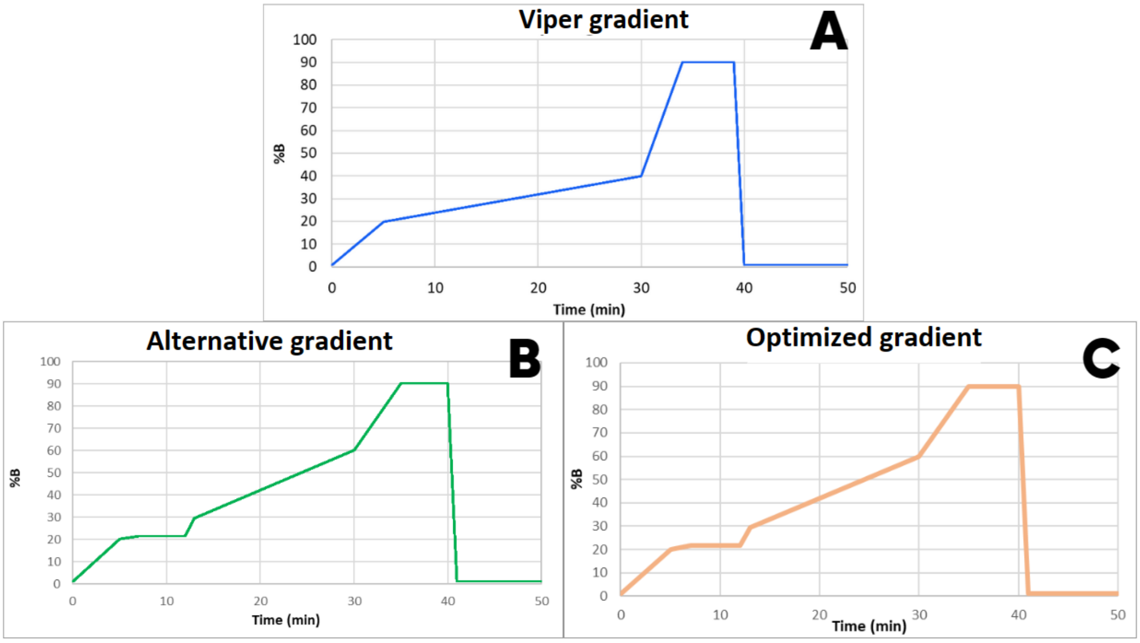

Figure S1: Different gradients that were applied during the gradient optimization phase performed in this study. ***A)*** *the "viper gradient" that was applied during prior research. This gradient had a linear increase of mobile phase B from 1% to 20% between 0.01 and 5 minutes. At 5 minutes there was a gradual linear increase of %B from 20% to 40% which took 25 minutes. At 30 minutes, the %B was sharply increased from 40 to 90%B in 4 minutes, while isocratic separation was obtained at 90%B for 5 minutes. At 39 minutes, %B was lowered back to 1% in one minute and the column was lastly equilibrated for 10 minutes at 1% B.* ***B)*** *the alternative gradient. This gradient was also tested to see if separation could be improved where the elution of SVMPs and SVSPs would be expected (roughly between 15 and 13 minutes). There was a linear increase of mobile phase B from 1% to 20% between 0.01 and 5 minutes. At 5 minutes there was a gradual increase of %B from 20% to 21.6% in 2 minutes, which was followed by an isocratic separation at 21.6%B for 5 minutes. At 12 minutes there was a small difference compared to the optimized gradient; the sharp increase that took one minute was from 21.6% to 30%B (compared to 29.6%B for the optimized gradient). In this gradient, there was also a sharp increase in %B from 30 to 40 %B starting at 13 minutes that took 1 minute. After this sharp increase, there was a slower linear increase of 11 minutes from 40 to 60%B. At 35 minutes there was another sharp increase that took one minute from 60 to 90%B, followed by isocratic separation from 36 to 40 minutes at 90%B. Similar to the optimized gradient, %B was then lowered back to 1% in one minute and the column was lastly equilibrated for 9 minutes at 1% B.* ***C)****, the final optimized gradient, which had a linear increase of mobile phase B from 1% to 20% between 0.01 and 5 minutes. At 5 minutes there was a gradual increase of %B from 20% to 21.6% in 2 minutes, which was followed by an isocratic separation at 21.6%B for 5 minutes. At 12 minutes there was a sharp increase from 21.6% to 29.6%B that took one minute. 13 minutes into the gradient, the %B was linearly increased in 17 minutes to reach 60%B. At 30 minutes, the %B was linearly increased to 90%B in 5 minutes, followed by an isocratic separation between 35 and 40 minutes. In 1 minute, %B was lowered back to 1% and the column was lastly equilibrated for 9 minutes at 1% B.*

Supporting Information: Qualitative profiling of venom toxins in the venoms of several *Bothrops* species using high throughput venomics and coagulation bioassaying

Based on findings of a previous study on *Bothrops* venoms [3], it was expected that the *Bothrops* venoms would be dominated by metalloproteinases, serine proteases and PLA_2s. Based on hydrophobicity, PLA_2s would elute first, while serine proteases and metalloproteinases would expectedly have a similar retention window at longer retention times. Based on the first HPLC-UV chromatograms using the original "viper" method, it was determined that the PLA_2s eluted between 8 and 10 minutes while the metalloproteinases and serine proteases eluted between 23 to 35 minutes for both *Bothrops* venoms analyzed.

Figure 2 shows that there is a significant amount of time in the gradient where no relevant peaks eluted for both *B. Jararaca* and *B. Neuwiedi* venoms when the viper gradient was used.

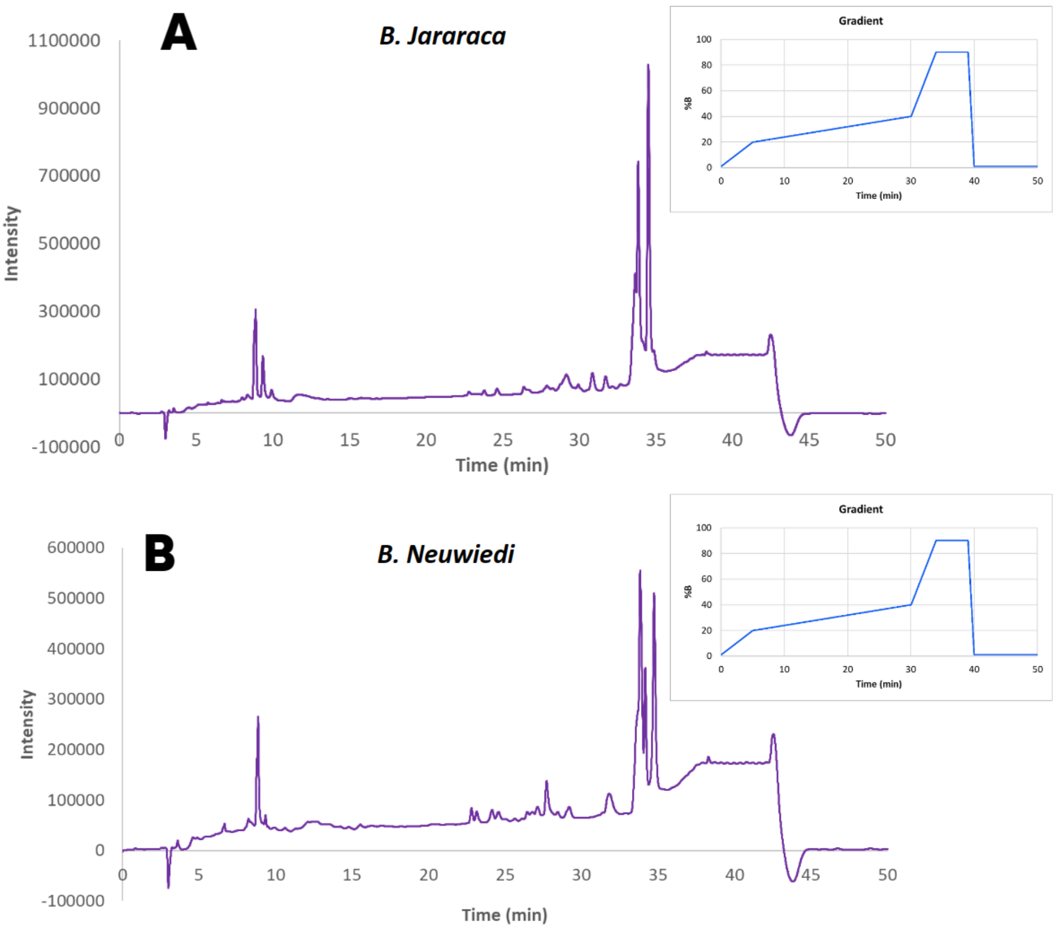

Figure S2: HPLC-UV chromatograms measured at 220nm of B. Jararaca venom in figure: ***A)*** *and of B. Neuwiedi venom in figure:* ***B)*** *using the original viper gradient program (19). This gradient had a linear increase of mobile phase B from 1% to 20% 0.01 and 5 minutes. At 5 minutes there was a gradual linear increase of %B from 20% to 40% which took 25 minutes. At 30 minutes, the %B was sharply increased from 40 to 90%B in 4 minutes, while isocratic separation was obtained at 90% B for 5 minutes. At 39 minutes, %B was lowered back to 1% in one minute and the column was lastly equilibrated for 10 minutes at 1% B.*

To improve the separation of mostly the SVMPs and SVSPs in the Bothrops venoms to be analyzed, the gradient was altered. The UV chromatograms using an altered "alternative" more optimal gradient and the final optimized gradient for the separation of the *B. Jararaca* venom can be seen in Figure 3.

Supporting Information: Qualitative profiling of venom toxins in the venoms of several *Bothrops* species using high throughput venomics and coagulation bioassaying

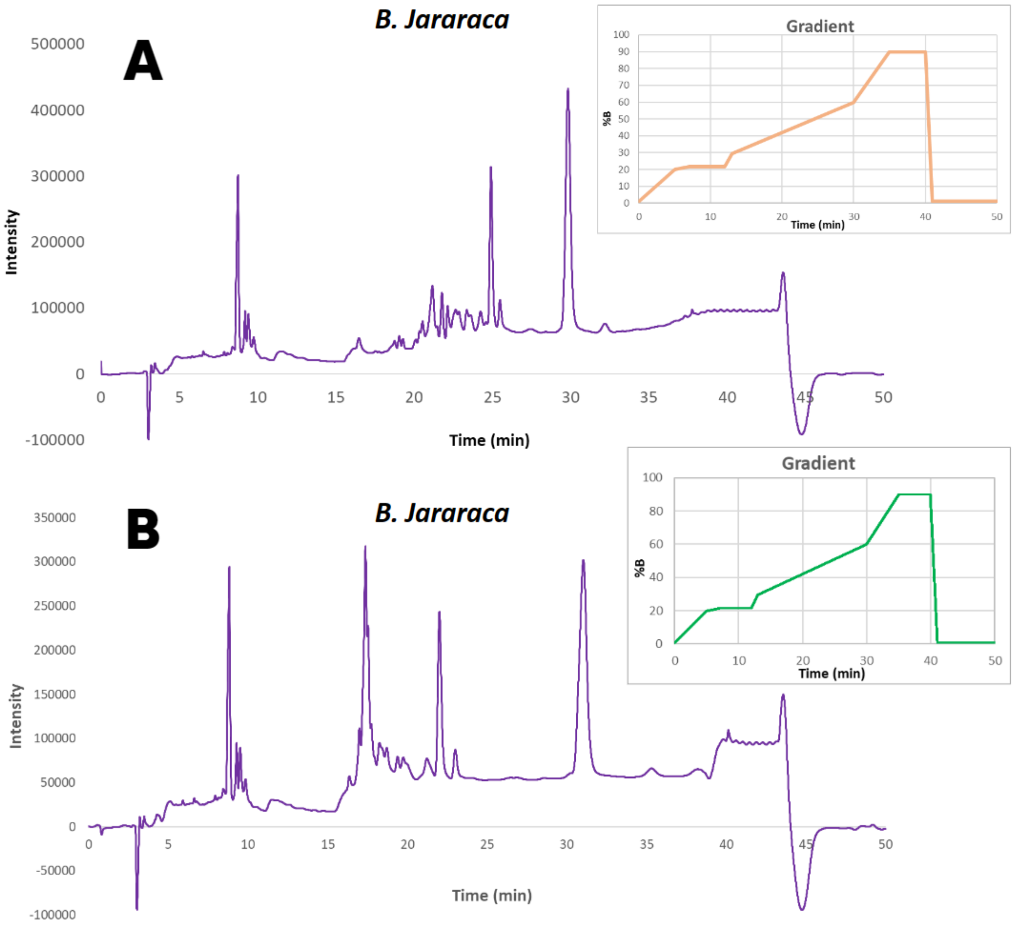

Figure S3: HPLC-UV chromatograms measured at 220nm of B. Jararaca venom for the optimized gradient: ***A)*** *and the alternative gradient:* ***B)*** *obtained during the gradient optimization experiments. The final optimized gradient,* ***A****), had a linear increase of mobile phase B from 1% to 20% between 0.01 and 5 minutes. At 5 minutes there was a gradual increase of %B from 20% to 21.6% in 2 minutes, which was followed by an isocratic separation at 21.6%B for 5 minutes. At 12 minutes there was a sharp increase from 21.6% to 29.6%B that took one minute. 13 minutes into the gradient the %B was linearly increased in 17 minutes to reach 60%B. At 30 minutes, the %B was linearly increased to 90%B in 5 minutes, followed by an isocratic separation between 35 and 40 minutes. In 1 minute, %B was lowered back to 1% and the column was lastly equilibrated for 9 minutes at 1% B. The alternative gradient is visualized in figure* ***B)****. There was a linear increase of mobile phase B from 1% to 20% between 0.01 and 5 minutes. At 5 minutes there was a gradual increase of %B from 20% to 21.6% in 2 minutes, which was followed by an isocratic separation at 21.6%B for 5 minutes. At 12 minutes there is a small difference compared to the optimized gradient; the sharp increase that took one minute in this case is from 21.6% to 30% B (compared to 21.6%B to 29.6%B for the optimized gradient). In this gradient, there was also a sharp increase in %B from 30 to 40 %B starting at 13 minutes that took 1 minute. After this sharp increase, there was a slower linear increase of 11 minutes from 40 to 60%B. At 35 minutes there was another sharp increase that took one minute from 60 to 90%B, followed by isocratic separation from 36 to 40 minutes at 90%B. Like the optimized gradient, %B was then lowered back to 1% in one minute and the column was lastly equilibrated for 9 minutes at 1% B.*

Resolution and peak separation using the viper gradient was improved by increasing elution time at the %B where toxins were expected to elute. The elution time was increased without increasing the analysis time by cutting out parts of the gradient where no relevant toxins seemed to elute (e.g., a jump from 21.6%B to 29.6%B in 1 minute for the optimal gradient). This resulted in an increased elution time for parts of the gradient where compounds of interest did

elute, which increased separation resolution of the peaks. An example is the plateau at 21.6% between 7 and 12 minutes for the separation of many of the PLA_2s. For the separation of many of the SVMPs and SVSPs, the %B was increased at a less steep slope, to elongate the part of the gradient where the toxins seemed to elute. As is depicted in Figure 3 these toxin families seemed to roughly elute between 23 and 35 minutes, while in the optimized gradient these toxin families eluted between 15 and 32 minutes (Figure 3). At 90% B no compounds seemed to elute, but this was kept in the program for flushing out the system. These chromatographic findings were similar for the experiments performed on *B. Neuwiedi* (Figure 4).

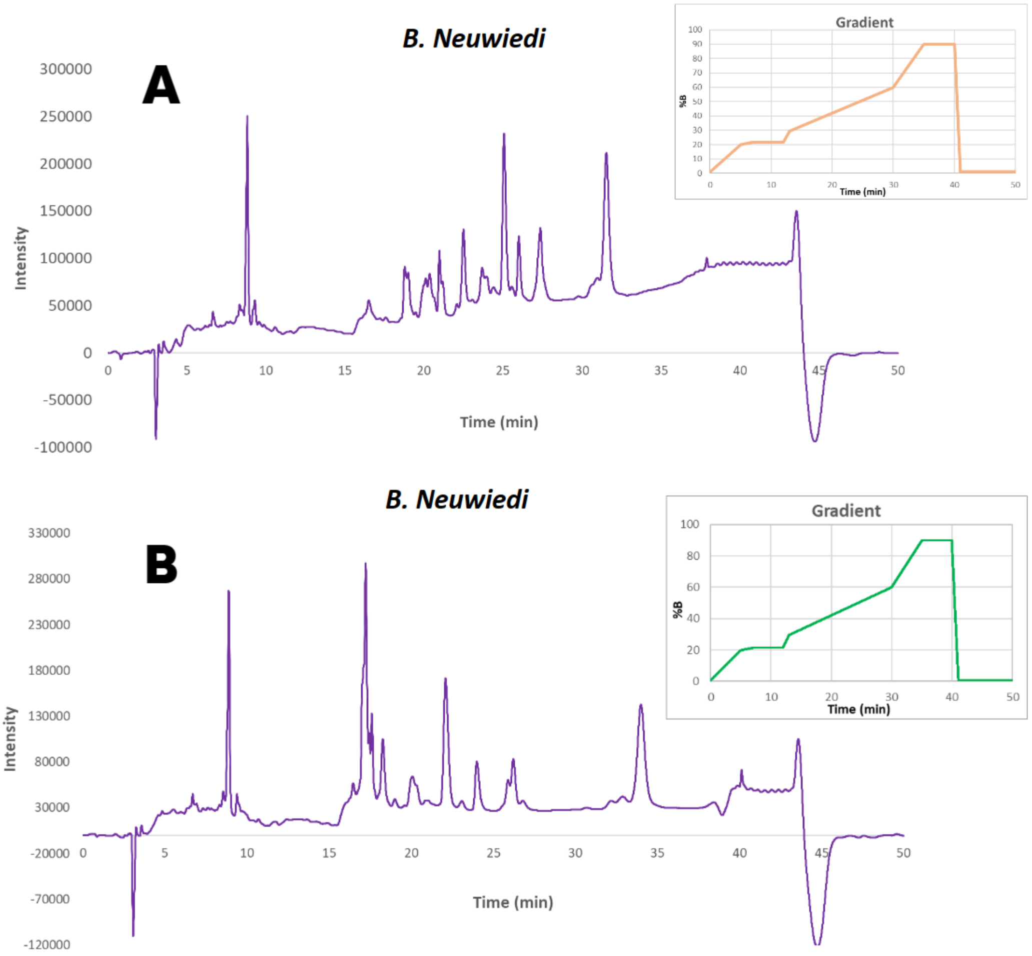

Figure S4: HPLC-UV chromatograms measured at 220nm of B. Neuwiedi venom for the optimized gradient: ***A)*** *and the alternative gradient:* ***B)*** *obtained during the gradient optimization experiments. The final optimized gradient,* ***A****), which had a linear increase of mobile phase B from 1% to 20% between 0.01 and 5 minutes. At 5 minutes there was a gradual increase of %B from 20% to 21.6% in 2 minutes, which was followed by an isocratic separation at 21.6%B for 5 minutes. At 12 minutes there was a sharp increase from 21.6% to 29.6%B that took one minute. 13 minutes into the gradient the %B was linearly increased in 17 minutes to reach 60%B. At 30 minutes, the %B was linearly increased to 90%B in 5 minutes, followed by an isocratic separation between 35 and 40 minutes. In 1 minute, the %B was lowered back to 1% and the column was lastly equilibrated for 9 minutes at 1% B. The alternative gradient is visualized in figure* ***B)****. There was a linear increase of mobile phase B from 1% to 20% between 0.01 and 5 minutes. At 5 minutes there was a gradual increase of %B from 20% to 21.6% in 2 minutes, which was followed by an isocratic separation at 21.6%B for 5 minutes. At 12 minutes, there is a small difference compared to the optimized gradient; the sharp increase that took one minute in this case was from 21.6% to 30%B (compared to 21.6%B to 29.6%B for the optimized gradient). In this gradient, there was also a sharp increase in %B from 30 to 40 %B starting at 13 minutes that took 1 minute. After this sharp increase, there was a slower linear increase of 11 minutes from 40 to 60%B. At 35 minutes there was another sharp increase that took one minute from 60 to 90%B, followed by isocratic separation from 36 to 40 minutes at 90%B. Like the optimized gradient, the %B was then lowered back to 1% in one minute and the column was lastly equilibrated for 9 minutes at 1% B.*

Supporting Information: Qualitative profiling of venom toxins in the venoms of several *Bothrops* species using high throughput venomics and coagulation bioassaying

References

3. Gren, E.C.K.; Kitano, E.S.; Andrade-Silva, D.; Iwai, L.K.; Reis, M.S.; Menezes, M.C.; Serrano, S.M.T. Comparative Analysis of the High Molecular Mass Subproteomes of Eight Bothrops Snake Venoms. *Comp Biochem Physiol Part D Genomics Proteomics* **2019**, *30*, 113–121, doi:10.1016/j.cbd.2019.01.012.

17. Slagboom, J.; Mladić, M.; Xie, C.; Kazandjian, T.D.; Vonk, F.; Somsen, G.W.; Casewell, N.R.; Kool, J. High Throughput Screening and Identification of Coagulopathic Snake Venom Proteins and Peptides Using Nanofractionation and Proteomics Approaches. *PLoS Negl Trop Dis* **2020**, *14*, 1–26, doi:10.1371/journal.pntd.0007802.

29. Sousa, L.F.; Freitas, A.P.; Cardoso, B.L.; Del-Rei, T.H.M.; Mendes, V.A.; Oréfice, D.P.; Rocha, M.M.T.; Prezoto, B.C.; Moura-da-Silva, A.M. Diversity of Phospholipases A2 from Bothrops Atrox Snake Venom: Adaptive Advantages for Snakes Compromising Treatments for Snakebite Patients. *Toxins (Basel)* **2022**, *14*, 543, doi:10.3390/toxins14080543.
36. Calvete, J.J.; Sanz, L.; Pérez, A.; Borges, A.; Vargas, A.M.; Lomonte, B.; Angulo, Y.; Gutiérrez, J.M.; Chalkidis, H.M.; Mourão, R.H.V.; et al. Snake Population Venomics and Antivenomics of Bothrops Atrox: Paedomorphism along Its Transamazonian Dispersal and Implications of Geographic Venom Variability on Snakebite Management. *J Proteomics* **2011**, *74*, 510–527, doi:10.1016/j.jprot.2011.01.003.
37. Sousa, L.F.; Holding, M.L.; Del-Rei, T.H.M.; Rocha, M.M.T.; Mourão, R.H.V.; Chalkidis, H.M.; Prezoto, B.; Gibbs, H.L.; Moura-Da-silva, A.M. Individual Variability in Bothrops Atrox Snakes Collected from Different Habitats in the Brazilian Amazon: New Findings on Venom Composition and Functionality. *Toxins (Basel)* **2021**, *13*, doi:10.3390/toxins13110814.
38. Cedro, R.C.A.; Menaldo, D.L.; Costa, T.R.; Zoccal, K.F.; Sartim, M.A.; Santos-Filho, N.A.; Faccioli, L.H.; Sampaio, S. V. Cytotoxic and Inflammatory Potential of a Phospholipase A2 from Bothrops Jararaca Snake Venom Suely V. Sampaio, Eliane C. Arantes, Marco A. Sartim. *Journal of Venomous Animals and Toxins Including Tropical Diseases* **2018**, *24*, doi:10.1186/s40409-018-0170-y.
39. Sousa, L.F.; Nicolau, C.A.; Peixoto, P.S.; Bernardoni, J.L.; Oliveira, S.S.; Portes-Junior, J.A.; Mourão, R.H. V.; Lima-dos-Santos, I.; Sano-Martins, I.S.; Chalkidis, H.M.; et al. Comparison of Phylogeny, Venom Composition and Neutralization by Antivenom in Diverse Species of Bothrops Complex. *PLoS Negl Trop Dis* **2013**, *7*, doi:10.1371/journal.pntd.0002442.
40. Kini, R.M.; Clemetson, K.J.; Markland, F.S.; McLane, M.A.; Morita, T. *Toxins and Hemostasis: From Bench to Bedside*; Springer Netherlands, 2011; ISBN 9789048192953.
